# Restless Wanderers

**Published:** 1860-12

**Authors:** 


### RESTLESS WANDERERS.

We are moved to pity many times in meeting with a class of men who are seeking for, they know not what. They see evil in the world and sorrow; they see oppression and degradation, and while observing them, feel the more, in that they have experiences in the same directions; tearful, bitter, almost heart-breaking experiences, it may be, and in blindness and powerlessness they are groping about wearily and painfully for a remedy.

In all these, not a single man or woman is found who does not begin by attacking the present system of received religion. Most of them persuade themselves that they believe the Bible, and readily refer to it as confirmatory of their peculiar systems, but in every case, they will only consent that the holy book shall be interpreted according to some preconceived views of their own. They are quite willing to make the Bible their arbiter, the tribunal of last resort, but then they insist that they must have the interpretation of its meaning. Yet with all this, they are dissatisfied and unhappy; there is a feeling of unrest which is devouring them, and they will talk *ad infinitum* to everybody, inferring from admissions of the occasional good sentiments which they avow, a more or less implied assent to their whole system, and drawing some comfort therefrom, they arrive at the conclusion that the whole world is rapidly falling into their views; and soon fanaticism assumes its sway, to hurry them to still greater extremes, until they are dashed on the rocks of suicide, of lunacy, or of perdition.

All these people look sad; they are extremely excitable; they fire up on the instant; and in all, we never fail to see a degree of bitterness towards opponents, and especially is a bitterness exhibited towards ministers, and churches, and communities, in proportion as these appear thriving, prosperous, and happy. Nor is this all; the rich are their universal anvil; on it they pound most mercilessly. With them, the selfishness of the rich is an exhaustless theme; or, if they ever come to a conclusion, it is this, that if these same rich people would commit the distribution of their property to them, the millenium would come in a very few days; and while handling the money which they never had the capacity to earn or keep, they would be the happiest people on the face of the earth, and would thence assume that everybody else was prosperous and happy too; just as a short time before, they had concluded that everybody was poor, and wretched, and miserable, because they were so themselves.

We earnestly counsel any chance reader of this article who has no heart-warming and cheerful religious faith of his own, to disabuse himself of the notion that the whole world is going wrong, by simply taking a general, generous, and liberal view of any evangelical denomination of Christians, and note for himself, in conversation with any considerable number of them, if there is not a most implicit faith in the great general doctrines of religion, of repentance, faith, and a new life; of the forgiveness of sins, of spiritual holification, of a Saviour born, and of final restoration to the bosom of the great Father of us all. They feel no more doubt of these things, than they do of the shining of the sun on a cloudless day; and more, they are humble in that belief as to themselves, and merciful and loving and forbearing as to others who are out of their faith, in that they spend their time and their money cheerfully, gladly, if by any means they can bring others to the knowledge of the great salvation; and withal, they are happy in their faith, happy in their hope, happy in their labors, and happy in their liberalities. Restless wanderers! if you will not believe this, "COME AND SEE."

